# Acute kidney injury following treatment with CD19-specific CAR T-cell therapy in children, adolescent and young adult patients with B-cell acute lymphoblastic leukemia

**DOI:** 10.21203/rs.3.rs-3396661/v1

**Published:** 2023-10-06

**Authors:** Yonique P Petgrave, Subodh Selukar, Rebecca Epperly, Swati Naik, Noel DeLos Santos, Brandon M Triplett, Stephen Gottschalk, John Bissler, Aimee C Talleur

**Affiliations:** University of Tennessee College of Medicine: The University of Tennessee Health Science Center College of Medicine; St Jude Children’s Research Hospital; St Jude Children’s Research Hospital; St Jude Children’s Research Hospital; University of Tennessee College of Medicine: The University of Tennessee Health Science Center College of Medicine; St Jude Children’s Research Hospital; St Jude Children’s Research Hospital; University of Tennessee College of Medicine: The University of Tennessee Health Science Center College of Medicine; St Jude Children’s Research Hospital

**Keywords:** CAR T-cell therapy, acute kidney injury, pediatric, CD19-CAR

## Abstract

CD19-specific chimeric antigen receptor (CAR) T-cell therapy has shown promising disease responses in patients with high-risk B-cell malignancies. Treatment with CD19-CAR T-cell therapy is also associated with the risk of morbidity and mortality, primarily related to immune-mediated complications (cytokine release syndrome [CRS] and neurotoxicity [NTX]), infections, and end-organ dysfunction. Despite these well-described systemic toxicities, the incidence of post-CAR T-cell therapy acute kidney injury (AKI) in the children, adolescent and young adult (CAYA) patient population is largely unreported. The objectives of this study were to determine the incidence of AKI in CAYA patients with high-risk B-cell malignancies treated with CD19-CAR T-cell therapy, evaluate potential risk factors for developing AKI, and determine patterns of kidney function recovery. In this retrospective analysis of 34 CAYA patients treated with CD19-CAR T-cell at a single institution, we found a cumulative incidence of any grade AKI by day 30 post-infusion of 20% (n=7), with 4 cases being severe AKI (Stage 2–3) and one patient requiring kidney replacement therapy. All episodes of AKI developed within the first 14 days after receiving CAR T-cell therapy and 50% of patients with AKI recovered kidney function to baseline within 30 days post-infusion. No evaluated pre-treatment risk factors were associated with the development of subsequent AKI; there was an association between AKI and CRS and NTX. We conclude that the risk of developing AKI following CD19-CAR T-cell therapy is highest early post-infusion, with most cases of AKI being severe. Although most patients with AKI in our cohort had recovery of kidney function, frequent monitoring to facilitate early recognition and subsequent management of kidney complications after CD19-CAR T-cell therapy may reduce the severity of AKI in the CAYA patient population.

## Introduction

CD19-specific chimeric antigen receptor (CAR) T-cell therapy is a highly effective immunotherapy that is increasingly used for the treatment of children, adolescent and young adult (CAYA) patients with relapsed and/or refractory B-cell acute lymphoblastic leukemia (B-ALL)(1–5). After infusion, CAR T-cell activation and expansion leads to a release of cytokines which may lead to systemic side effects, including cytokine release syndrome (CRS) and neurotoxicity (NTX), now known as immune effector cell-associated neurologic syndrome (ICANS)(6–8). In the setting of these toxicities, multiorgan dysfunction may occur, including acute kidney injury (AKI)(9, 10).

Reports on AKI post-CAR T-cell therapy are limited. A few studies of adult patients treated with CD19-CAR T-cell therapy demonstrate a wide range of incidences of AKI, anywhere from 5–30%(9, 11, 12). These studies highlight the high association between AKI severity and the need for kidney replacement therapy, and increased mortality(13). Based on the existing literature, the etiology of AKI in adult patients is most likely multifactorial, including a lower pre-treatment glomerular filtration rate (GFR), and post-infusion development of CRS and/or NTX (12). AKI is even less well described in the CAYA patient population, with reports limited to early-phase clinical studies(14–16). Furthermore, the identification of AKI in CAYA patients may be underestimated due to the use of serum creatinine (sCr) as a biomarker of kidney function, which may be falsely low due to poor muscle mass, therefore overestimating kidney function(17–19).

Accurate diagnosis of AKI and identification of risk factors for developing AKI are necessary to further our understanding of this toxicity post-CAR T-cell therapy. Subsequently, this may facilitate preemptive mitigation and management strategies, thereby reducing the incidence of AKI and its subsequent adverse outcomes. In this study, we aimed to determine the incidence and severity of AKI in CAYA patients that received treatment with CD19-CAR T-cell therapy at a single tertiary care cancer center. We also investigated possible associations between pre- and post-treatment patient and clinical parameters with the development of AKI, to identify potential predictive markers for AKI post-CAR T-cell therapy in this patient population.

## Materials and Methods

This is a retrospective review of CAYA patients (n = 34) with relapsed and/or refractory CD19-positive malignancy, treated with lymphodepleting chemotherapy (fludarabine and cyclophosphamide) and CD19-CAR T-cells (tisagenlecleucel or an institutional product [NCT03573700]) at St. Jude Children’s Research Hospital between October 2018-April 2021. Both CD19-CAR T-cell products utilize the CD19-specific single chain variable fragment (scFv) FMC63 and a 4–1BB costimulatory domain(20). Written informed consent/assent was obtained from all patients and/or legal guardians to receive treatment with lymphodepletion and CD19-CAR T-cell therapy, per institutional guidelines and the Declaration of Helsinki. This retrospective project was approved by the St. Jude institutional review board. Data were collected from a clinical database and retrospective medical record review. Collected data included patient demographics, disease- and treatment-related characteristics, pre- and post-infusion laboratory values, and post-infusion side effects. Data were divided into two time periods: pre-treatment (day – 30 to day 0) and post-treatment (day 1 to day 30 post-CAR T-cell infusion). Baseline blood pressure was determined using the average of all available blood pressure recordings from the pre-infusion period. Patients were defined as hypertensive pre infusion based on the use of antihypertensive medications at the time of infusion. CRS was defined by the ASTCT consensus criteria, including retrospective application of these criteria for those patients treated prior to the release of the ASTCT criteria(7). For NTX, prior to the adoption of the ASTCT guidelines (ICANS), NTX was graded using the common terminology adverse event criteria, in conjunction with a protocol-defined grading scheme.

Collected data on kidney function included all available creatinine and cystatin C levels from pre-treatment through post-infusion, with data collection stopping at the time of death, leukemic non-response to CAR, leukemic disease progression or receipt of subsequent therapy, including allogeneic hematopoietic cell transplantation (AlloHCT), which ever occurred first. Cystatin C was measured using the International Federation of Clinical Chemistry calibrated cystatin C(24). Baseline sCr level was defined as the measurement closest to, and preceding the start of, lymphodepleting chemotherapy. There were no missing data for baseline creatinine values. Additionally, the determined glomerular filtration rate (GFR) was documented from any available 24-hour creatinine clearance and Technetium 99 DTPA scans (Tech99) obtained during this same period. Estimated glomerular filtration rate (eGFR) using creatinine and cystatin C was calculated Chronic Kidney Disease Epidemiology Collaboration U25 Eq. (23). Post-infusion, when multiple creatinine values were available for a patient on a given day, the maximum daily value was utilized in the analysis. An attempt to further subdivide kidney function based was done by weeks post-CAR T-cell infusion, with last week rounded to day 30. Due to limited availability of cystatin C, the value closest to the timepoint of interest was utilized.

AKI was defined as a 1.5-fold or greater increase in sCr level from baseline within 30 days post-infusion, or requirement for kidney replacement therapy. AKI severity was graded using KDIGO (Kidney Disease: Improving Global Outcome) criteria such that: grade 1 = 1.5 - < 2-fold, grade 2 = 2 - < 3-fold, and grade 3 = ≥ 3-fold increase from baseline sCr(25). Of note, a decrease in urine output is also part of KDIGO grading but was not used in this study due to its retrospective nature. There is no standard definition of AKI by cystatin C; therefore, changes in eGFR used to determine cystatin C AKI were modeled off those used for creatinine-based AKI. AKI by cystatin C was categorized as mild or severe, based on a 50% or 70% reduction in GFR, respectively(26). Kidney recovery was defined as a return of creatinine to pre-CAR T-cell therapy baseline.

Patient baseline characteristics, laboratory values and accompanying toxicities were summarized by the median (range) or count (percent) and grouped by AKI presence. Time to any AKI in the 30 days after CAR T-cell infusion was summarized by the cumulative incidence function and calculated as 1-Kaplan-Meier estimate (there were no competing risks of death before AKI within 30 days). Patients without AKI were censored at 30 days or date of last data collection, whichever is earlier. Associations between AKI within 30 days and baseline characteristics were assessed by univariate logistic regression models, and associations between time to AKI and CRS or NTX were assessed by univariate Cox models with time-varying covariates (0 before occurrence of CRS or NTX, respectively, and 1 afterward). Except where otherwise-specified, analyses were performed at the infusion-level (only one patient had multiple infusions, and these two infusions were treated as independent). Analyses were performed using R 4.1 software. P-values and 95% confidence intervals are reported without adjustment for multiplicity and are to be considered exploratory.

## Results

During the study period, 34 patients with relapsed and/or refractory CD19-positive malignancy were treated with lymphodepleting chemotherapy and CD19-CAR T cells, for a total of 35 infusions. One patient received treatment with both products, with > 1 year and receipt of an AlloHCT occurring in between infusions, and therefore contributed to this study twice. Patient, disease, and treatment characteristics are shown in Table 1. Males accounted for 55.9% of the cohort. The median age at time of infusion was 9.7 years (range 1.8–23.6 years), with most participants identifying as White (73.5%). Almost all patients had B-ALL, with 1 patient having B-cell acute lymphoblastic lymphoma. Patients were heavily pretreated, with 28.6% of infusions occurring after previous AlloHCT. Prior to CAR T-cell therapy, 18 patients had a low disease burden (≤ 5% morphologic blasts in the marrow) and 17 patients had a high disease burden (> 5% blasts). The median morphological blast burden was 5.0% (range 0–98%). Hypertension before CAR T-cell therapy was noted in 5 participants. Post-infusion, CRS was seen after 60% of infusions (n = 21), with only 6 episodes being high grade (grades 3–4). Neurotoxicity occurred after 25.7% of infusions (n = 9), 4 of which were high grade (grades 3–4) (Table 1). Six patients required admission to the intensive care unit for the management of CAR T-cell related side effects.

**Figure 1:**
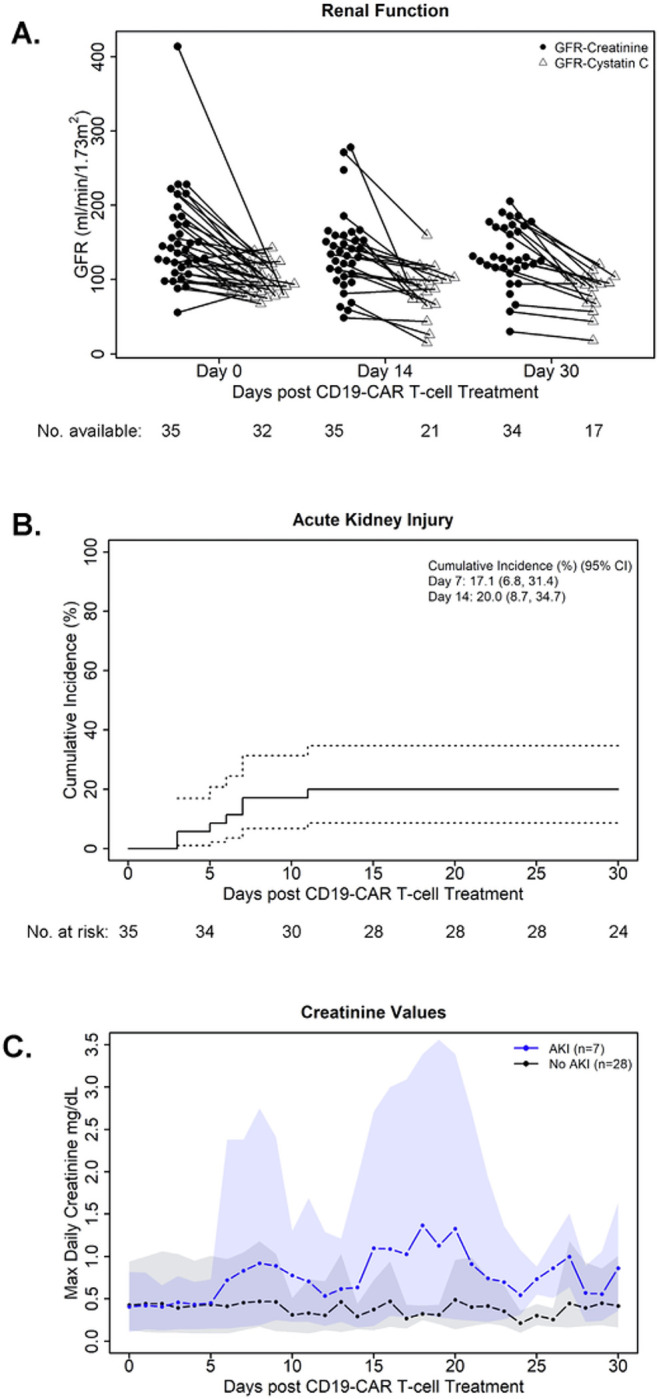
Renal function in CAYA patients after treatment with CD19-CAR T-cell Therapy. **(A)**Glomerular filtration rate (GFR) of patients at 3 times points, determined by serum creatinine and, in a subset of patients with available data, cystatin C. GFR was calculated using the Chronic Kidney Disease Epidemiology Collaboration U25 equation. Time points shown include: Day 0, Day 14 and Day 30. In most patients with paired GFR data by creatinine and cystatin C, GFR by creatinine was higher than by cystatin C. **(B)**The cumulative incidence (CI) of acute kidney injury (AKI) post CD19-CAR T-cell therapy, within 30 days post infusion. AKI was defined as 1.5-fold or greater increase in serum creatinine level from baseline. All patients developed AKI within 14 days after CAR T-cell infusion, with a 20% CI (95% CI [dotted lines]: 8.7 – 34.7). **(C)** Summary of daily maximum creatinine within 30 days of CAR T-cell infusion by AKI group (blue, AKI; black, no AKI). Lines represent the average across patients within a group and shading corresponds to the range (min-max) within a group. For the first 3 days after infusion, all patients have available creatinine. For later days, the number of patients with available measurements on a given day ranged with later days typically having fewer available measurements (AKI group, range: 3–7; no AKI group, range: 3–27 values).

All patients had a normal GFR before CAR T-cell therapy, including by 24hr urine collection for creatinine clearance (n = 17 patients; median GFR 155 ml/min/1.73m^2^, range 71–280) or by Tech99 (n = 19; median GFR 125 ml/min/1.73m^2^, range 60–207) (Table 1). One patient had both studies performed before infusion. The median baseline sCr (n = 35) before CAR T-cell therapy was 0.3 mg/dl (range 0.1–0.9), giving a calculated baseline eGFR of 142 ml/min/1.73m^2^ (range 56–414). The median baseline cystatin c (n = 32) was 0.8 mg/L (range 0.6–1.1), with a calculated eGFR of 95 ml/min/1.73m^2^ (range 68–143). Based on combined creatinine-cystatin C equation (n = 32), the median eGFR was 124 ml/min/1.73m^2^ (range 68–247). Post-infusion, GFR based on sCr and cystatin C was evaluated at days 14 and 30. We found that across time points, patients frequently had higher eGFR based on sCr compared to cystatin C (Fig. 1A).

After treatment with CD19-CAR T-cell therapy, 7 patients developed AKI by sCr, resulting in an overall cumulative incidence (CI) of AKI of 20% by day 30 (Fig. 1B). Severe AKI (Stage 2 or 3) occurred after 4 infusions (11.4%). Most patients (n = 6) developed AKI within the first 7 days post infusion, with all episodes of AKI beginning within 14 days post infusion (Fig. 1B). Of note, most patients with AKI (n = 6) experienced a peak in creatinine within 14 days of infusion (≤ 7 days, n = 3), with only one patient having a peak sCr in the third week post infusion. This compares to patients that did not develop AKI, in which sCr levels remained relatively stable throughout the post-infusion period (Fig. 1C). While most patients with AKI were able to be managed conservatively, 1 patient required kidney replacement therapy due to fluid overload and severe electrolyte derangements. Among the 7 patients with AKI, 5 recovered kidney function and 2 died prior to recovery of kidney function. Of those that recovered kidney function, 3 recovered within 30 days post infusion (Fig. 1C). Of note, within our dataset cystatin C was not uniformly measured across patients. This precluded the use of cystatin C in a time-to-occurrence model to establish the cumulative incidence of AKI by cystatin C in this patient cohort. From available cystatin C data, a total of 4 patients had AKI by cystatin C. This included 3 patients that also had AKI by sCr and 1 additional patient that did not have AKI by sCr. Among the 4 patients with cystatin C based AKI, 3 were classified as mild and 1 as severe.

**Figure 2:**
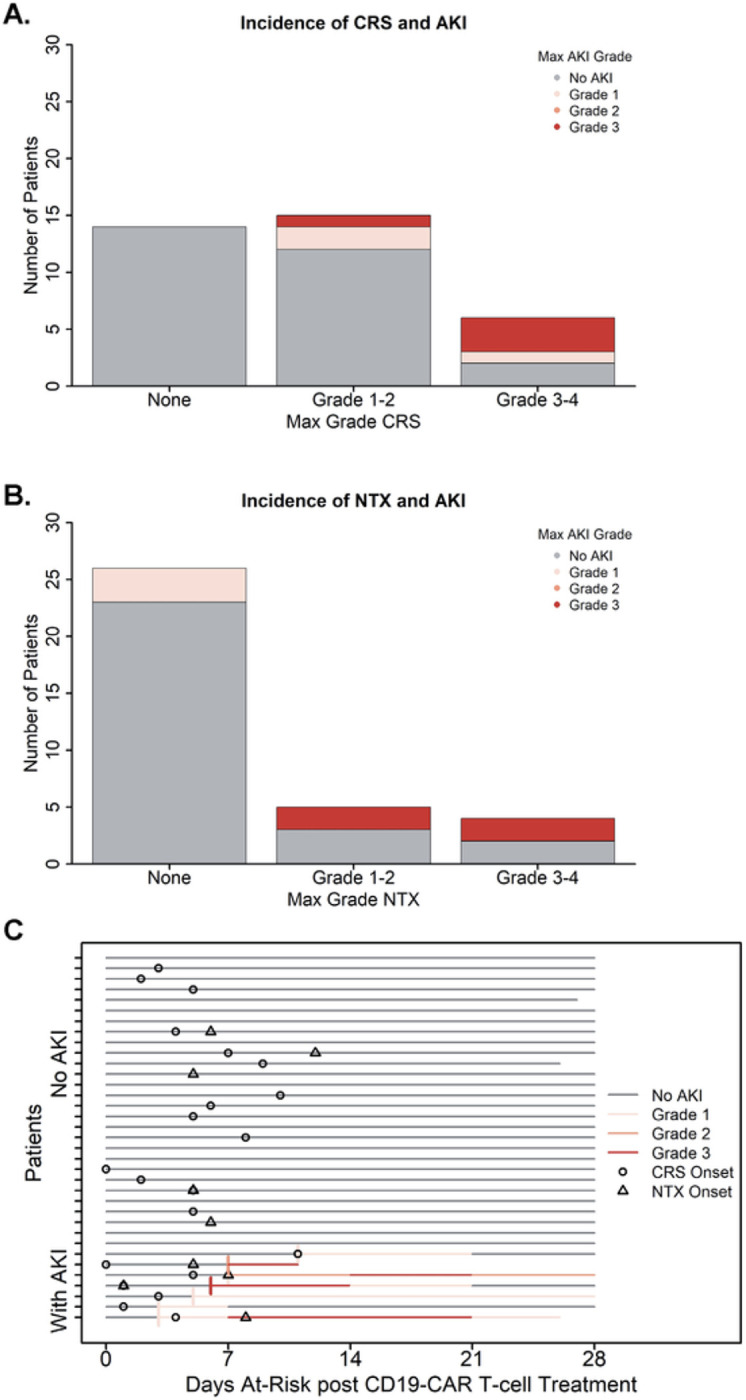
Association between development of AKI and post CD19-CAR T-cell immune mediated side effects. Incidence of CAYA patients experiencing acute kidney injury (AKI) after CD19-CAR T-cell therapy in relation to presence of immune mediated side effects (cytokine release syndrome, CRS; and neurotoxicity, NTX). AKI severity was graded using KDIGO (Kidney Disease: Improving Global Outcome) criteria. Patients with AKI are presented in the colored bars, by max AKI grade; patients without AKI are accounted for in the gray bar. (A) Distribution of patient by highest grade CRS. All patients with AKI also had CRS, with AKI being more prevalent in patients with higher-grade CRS. (B) Distribution of patient by highest grade NTX. All patients with higher grade AKI also had NTX. (C) Swimmers plot depicting the course of AKI for each patient, with each lane representing a single patient. AKI onset is shown by the start of a colored bar; after onset, the max AKI grade is shown in week (7-day) intervals. Onset of immune-mediated side effects are denoted using symbols (circle, CRS; triangle, NTX).

We next sought to investigate associations between pre- and post-treatment characteristics and the development of AKI after CD19-CAR T-cell therapy. None of the evaluated pre infusion characteristics had a statistically significant relationship with the development of AKI post-infusion (Table 2). Post infusion, there was a relationship between CRS, NTX and AKI, such that the presence of CRS and/or NTX correlated with a higher rate of AKI on univariate analysis (CRS, HR 6.24 [95% CI: 1.15–33.96, p = 0.034]; NTX, HR 4.24 [95% CI 0.74–24.12, p = 0.104]). Additionally, those with more severe CRS/NTX also had more severe AKI (Figs. 2A,B). Notably, all 7 patients with AKI also had CRS, with onset of AKI occurring either at the same time or after onset of CRS for most patients (n = 6). Among the four patients who developed AKI and NTX, the timing of AKI in relation to onset of NTX was similar, with 3 of the 4 patients developing AKI at the same time or after onset of NTX (Fig. 2C).

## Discussion

Although the use of CD19-CAR T-cell therapy has revolutionized the treatment paradigm for CAYA patients with relapsed/refractory B-cell ALL(1, 4, 28), it may be associated with risk of toxicity related morbidity and mortality. Importantly, these toxicities are associated with risk of multi-organ dysfunction and/or failure, including AKI(29, 30). While AKI post-CAR T-cell therapy has been described in several reports of adult patients, these data are lacking in the CAYA population(15). In our study of 34 CAYA patients treated with CD19-CAR T-cell therapy, we found an incidence of AKI based on creatinine of 20%, with most episodes being more severe. Additionally, all instances of AKI occurred within the initial 14 days post-infusion, and there was a relationship between the presence of CRS/NTX and the development of AKI, particularly severe CRS/NTX.

Our study found an incidence of AKI in pediatric CD19-CAR T-cell recipients similar to the limited previously published data (15). Importantly, our data highlights that the majority of CAYA patients who developed AKI had a more severe form, and that AKI is most likely to develop early after CAR T-cell therapy. While half of the patients in our cohort had a return of kidney function to baseline before day 30 post-infusion, one patient did require kidney replacement therapy, and the long-term impact on kidney function is unknown. These data highlight the significant risk of AKI in the CAYA patient population and should prompt close monitoring of kidney function in the early post-infusion period to facilitate management changes aimed at minimizing kidney injury. Given the known increased morbidity and mortality associated with AKI in the post-transplant population(35–38), strategies to mitigate the risk of AKI should be implemented in patients undergoing CAR T-cell therapy as well.

Patients treated with CAR T-cell therapy may be predisposed to the development of AKI, including due to the receipt of multiple prior treatment regimens, prior episodes of AKI, and lower pre-treatment GFR(12, 18, 31). While our study was not able to identify pre-treatment variables that were associated with risk of developing AKI post-CAR T-cell therapy, pre-treatment disease burden did show a large, estimated effect size, but with a wide variability. While our sample size may limit our power to determine such an association, this result is consistent with previous reports in CAYA patients(15). However, this differs from data of adult patients where pre-treatment factors such as prior AlloHCT and lower baseline GFR portended a higher risk of developing AKI. This difference may relate to patient age, and the expected decline in kidney function and other age-related physiologic changes(32). Post-CAR T-cell therapy, additional risk factors for the development of AKI may be present, including exposure to nephrotoxic agents, the development of tumor lysis syndrome, infection or, particularly for patients treated with CAR T-cells, the development of systemic inflammation(33). Consistent with previous reports in both adults and CAYA patients(6, 15, 16), we found a strong association between CRS and NTX and the rate of AKI. Biologically, this is a rational association given that CRS includes hypotension and vasodilatory shock, which may lead to reduced kidney perfusion and subsequent kidney injury(34).

Tools such as cystatin C and other biomarkers may assist in earlier detection of AKI, and therefore allow for improved management and reduction in prevalence of severe AKI. Serum cystatin C is a sensitive marker of AKI in a variety of patient populations(39, 40), and in an oncologic CAYA patient population, cystatin C has been described as a potential tool to detect eGFR changes earlier than sCr after an insult, with superior sensitivity for detecting minor changes in kidney function(38). Cystatin C has also been readily identified as a more sensitive marker of kidney function in the CAYA patient population, as these patients tend to have lower muscle mass, thus leading to overestimation of GFR with creatinine-based equations(17, 41). However, there have been caveats to its use in the presence of rapid cell turnover, as well as steroid use. To our knowledge, no study has looked at cystatin C based AKI in patients treated with CAR T-cell therapy. Our data on cystatin C are limited by sample size, variable monitoring patterns and, anecdotally, increased use of cystatin C testing in patients suspected to have a change in kidney function. However, our data highlight that in this CAYA patient population, cystatin C based eGFR trended lower than sCr based eGFR both pre- and post-CAR T-cell infusion. Additionally, we were able to detect AKI in 1 patient by cystatin C that was not apparent by sCr. In future prospective studies, routine monitoring of cystatin C may provide more robust data to determine the utility of using cystatin C in assessing kidney function and potentially risk stratifying for AKI in this patient population.

In conclusion, our study identified that CAYA patients undergoing CD19-CAR T-cell therapy have a high incidence of AKI post-infusion, particularly in the initial weeks after treatment. We also demonstrate a strong association between the development of AKI and presence of CRS or NTX. While many patients recovered their kidney function, the long-term effects of this injury are unknown. As the experience in CAR T-cell therapy grows for various malignancies, larger studies are needed to risk stratify patients for specific organ toxicities, including kidney injury. Furthermore, investigation into the utility of other biomarkers of kidney function such as cystatin C, may more readily identify AKI in this population, leading to earlier interventions intended to reduce morbidity and mortality.

## Figures and Tables

**Table 1:** Patient Characteristics

	No AKI	AKI	Overall#

**Patient-Level Characteristics**	**N = 27**	**N = 7**	**N = 34**

Sex	15 (78.9)	4 (21.1)	19
Male	12 (80.0)	3 (20.0)	15
Female			

Race	21 (84.0)	4 (16.0)	25
White	4 (66.7)	2 (33.3)	6
Black	2 (66.7)	1 (33.3)	3
Other			

Primary Diagnosis	27 (81.8)	6 (18.2)	33
B-ALL	0 (0.0)	1 (100.0)	1
Lymphoma			

**Infusion-Level Characteristics**	**N = 28**	**N = 7**	**N = 35**

**Pre-Infusion**			

Age (years)	9.4 (1.8–23.6)	11.8 (1.8–20.4)	9.7 (1.8–23.6)

BMI	18.6 (13.5–43.2)	20.8 (15.6–41.2)	18.8 (13.5–43.2)

Disease Burden (bone marrow)*	16 (88.9)	2 (11.1)	18
Low: ≤5%	12 (70.6)	5 (29.4)	17
High: >5%			

Pre-infusion GFR ml/min/1.73m2	125.0 (60.0–207.0)	128.5 (105.0–145.0)	125.0 (60.0–207.0)
GFR-Tech99 (n = 19)	155.0 (77.0–212.0)	146.0 (71.0–280.0)	155.0 (71.0–280.0)
GFR-24Cr (n = 17)			

Prior AlloHCT	19 (76.0)	6 (24.0)	25
No	9 (90.0)	1 (10.0)	10
Yes			

Prior Hypertension (HTN)^%^	25 (83.3)	5 (16.7)	30
No	3 (60.0)	2 (40.0)	5
Yes			

**Post-Infusion**			

Max Grade CRS	14 (100.0)	0 (0.0)	14
0	12 (80.0)	3 (20.0)	15
1–2	2 (33.3)	4 (66.7)	6
3–4			

Max Grade NTX	23 (88.5)	3 (11.5)	26
0	3 (60.0)	2 (40.0)	5
1–2	2 (50.0)	2 (50.0)	4
3–4			

Numerical data are presented as n(%) or median (range)

# 34 unique patients received a total of 35 CD19-CAR T-cell infusions;

* morphologic blast percent in the bone marrow prior to CAR T-cell therapy;

^ Obtained within 2 weeks prior to start of CAR T-cell therapy by Tech99 (n = 19) and/or 24-hour creatinine clearance (n = 17);

% presence of HTN within 30 days prior to CAR T-cell infusion; AKI, acute kidney injury; B-ALL, B-cell acute lymphoblastic leukemia; BMI, body mass index; GFR, glomerular filtration rate; AlloHCT, allogeneic hematopoietic cell transplantation; CRS, cytokine release syndrome; NTX, neurotoxicity.

**Table 2:** Univariable analysis of risk factors associated with the development of AKI within 30 days after CD19-CAR T-cell therapy

Variable#	OR (95% CI)	P**
Sex: Female* vs. Male	1.00 (0.19, 5.88)	1.000
Race: White* vs. Other	2.75 (0.44, 16.24)	0.264
Age (years)	1.01 (0.87, 1.16)	0.917
BMI	1.10 (0.99, 1.23)	0.079
Prior AlloHCT: No* vs. Yes	0.35 (0.02, 2.51)	0.324
Disease Burden:^&^ Low* vs. High	3.33 (0.60, 26.18)	0.171
Prior Hypertension^%^: No* vs. Yes	3.33 (0.37, 25.98)	0.261
GFR-Tech99^^^	1.00 (0.96, 1.03)	0.821
GFR-24Cr^^^	1.01 (0.98, 1.03)	0.650
Post Infusion Variable	HR (95%CI)	p
Cytokine Release Syndrome	11.39(1.31–99.12)	0.028
Neurotoxicity	12.45 (2.45–62.38)	0.002

# n = 35;

** Likelihood ratio test;

* reference group;

& morphologic blast percent in the bone marrow prior to CAR T-cell therapy (low: ≤5%, high: >5%);

% presence of HTN within 30 days prior to CAR T-cell infusion;

^ obtained within 2 weeks prior to start of CAR T-cell therapy by Tech99 (n = 19) and/or 24-hour creatinine clearance (n = 17); AKI, acute kidney injury; BMI, body mass index; AlloHCT, allogeneic hematopoietic cell transplantation; GFR, glomerular filtration rate.

## Highlights

- Children, adolescent and young adult patients are at risk for developing AKI after treatment with CD19-CAR T-cell therapy.
- Risk of developing AKI is highest within the first 14 days after CD19-CAR T-cell therapy.
- Development of AKI is associated with the development of cytokine release syndrome and neurotoxicity.
